# Protein PEGylation: Navigating Recombinant Protein Stability, Aggregation, and Bioactivity

**DOI:** 10.1155/2022/8929715

**Published:** 2022-07-25

**Authors:** Lindiwe Khumbuzile Zuma, Nothando Lovedale Gasa, Xolani Henry Makhoba, Ofentse Jacob Pooe

**Affiliations:** ^1^Discipline of Biochemistry, School of Life Sciences, University of KwaZulu-Natal, Westville Campus, Westville, 3629 KwaZulu-Natal, South Africa; ^2^Department of Biochemistry and Microbiology, University of Fort Hare, Alice Campus, Alice, 5700 Eastern Cape, South Africa

## Abstract

Enzymes play a powerful role as catalysts with high specificity and activity under mild environmental conditions. Significant hurdles, such as reduced solubility, reduced shelf-life, aggregate formation, and toxicity, are still ongoing struggles that scientists come across when purifying recombinant proteins. Over the past three decades, PEGylation techniques have been utilized to significantly overcome low solubility; increased protein stability, shelf-life, and bioactivity; and prevented protein aggregate formation. This review seeks to highlight the impact of PEG-based formulations that are significantly utilized to obtain favourable protein physiochemical properties. The authors further discuss other techniques that can be employed such as coexpression studies and nanotechnology-based skills to obtaining favourable protein physiochemical properties.

## 1. Introduction

**Figure 1:**
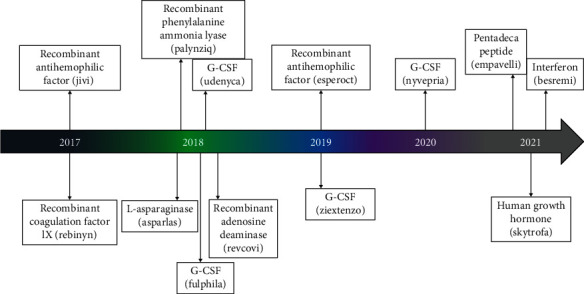
Recent FDA approved PEGylated drugs (2017-2021).

The physical or chemical interaction between polyethylene glycol (PEG) and bioactive molecules and nanoparticles is defined as PEGylation [1]. PEGylation has been associated with therapeutic level benefits such as increasing protein solubility, thermal, and chemical stability; reducing toxicity; increasing proteolysis resistance; increasing hydrodynamic volume; and reducing protein aggregation [2]. Furthermore, various studies previously revealed that PEG conjugation into proteins has never changed protein structure. Thus, this highlights an unquestionable fact that PEGylation is an interesting topic and vital for applications in several fields of study including the pharmaceutical industry (Figure 1) [3]. There are several studies illustrating the effective application of PEGylation such as its application in extending the half-life (from 13.6 minutes to 4.5 hours) of bovine serum albumin (BSA) comparatively to non-PEGylated nanoparticles resulting from PEGylation of poly (lactic-co-glycolic acid) (PLGA) nanoparticles that encapsulated bovine serum albumin (BSA) (size of 200 nm and efficiency entrapment of 48.6%) [4].

**Figure 2:**
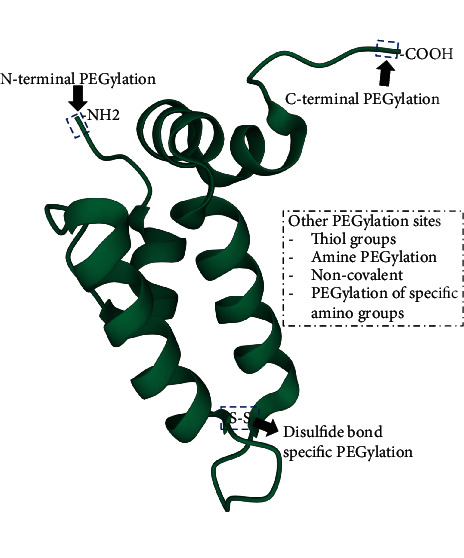
Demonstration of prospective PEGylation sites in proteins.

PEG has a noncharged, flexible, and hydrophilic backbone with only terminal sites which are accessible for interactions and functionalization. PEGylation processes are classified as noncovalent and covalent PEGylation processes regulated by the reaction used (Figure 2). Noncovalent PEGylation (recent scarcely employed) makes use of hydrophobic and ionic interactions to form complexes linking the polymer or protein. The nonspecific PEGylation method previously used by David and Abuchowsky in the late 1970s demonstrated that PEG conjugation on proteins can subsequently reduce protein aggregation and proteolysis and extend protein shelf-life [5]. Noncovalent PEGylation is referred to as the first generation PEGylation which typically utilizes amine conjugation. Furthermore, the main objective of the first-generation PEGylation involves irreversible conjugation [6]. There are limitations of widespread use of this method due to the removal of PEG coating.

The first generation PEGylation has evolved into a second-generation PEGylation which involves a site-specific PEGylation method, thus increases PEGylation specificity between PEG molecules conjugation with particular moieties in the protein [7]. In the covalent PEGylation method, stable chemical bonds are formed conceivable site-specific [8]. The covalent technique is most desirable and can be cost-effective. PEGylation through thiol, N-terminal, enzymatic, and histidine tags is some of the methods used to conduct site-specific PEGylation (Figure 2) [9]. The main pathway of site-specific PEGylation is constantly reversible conjugation, which does not inhibit conjugate activity. Therefore, the cleavable linkages are utilized to allow temporal attachment of PEG molecules, and the conjugates can be released accordingly at a specific time schedule [6, 10].

However, a covalent technique has some limitations; not always feasible and may require the highest development time. Furthermore, the several target specific sites present in the molecule could lead to the development of PEGylated species with varying modification degrees and isomerism position similarly to random PEGylation. The third generation PEGylation is sought to be developed to achieve higher potency and circulation half-life inflexible on fast-acting, site-specificity, and lower dosages [11]. Overall, this review illustrates the effects of PEGylation on protein stability and summarizes target amino acids for site-specific PEGylation and analytical methods used to characterize PEGylated proteins.

## 2. Effects of PEGylation on Protein Stability

Several reports have demonstrated the effectiveness of PEG towards protein conformational stability. Recently, studies and computational simulations showed efficiency of PEGylation process regarding alteration of protein conformational stability [5]. Protein stability broadly refers to stability against proteases, thermal stability, thermodynamic stability, and dissemination in a live attenuated, chemical, and structural stability [12]. Therefore, protein modifications such as protein PEGylation intending to enhance stability are preferably selected due to their ability to operate without disrupting protein secondary structures, irrespective of the PEG chain adopted conformation. However, PEGylation strategies have been shown to positively, negatively, or ineffectively affect protein stability. Previously, [13] discovered that the secondary structure motif is not specifically responsible for PEG to induce protein stability. Alternatively, the orientation PEG appeared to be the most influential factor inducing stability [14]. Furthermore, the study conducted by Abuchowski et al. (1977) proclaimed that amino acid side chains are available for conjugation [15]. Additionally, PEG conjugation of BSA showed an increase in proteolytic stability, thermal stability, and pH stability was observed. Therefore, their study influenced further understanding of the effects of different PEG (linear-PEG, branched-PEG, and non-PEG) polymers on the stability and conformation of many proteins. Still, similarly, the results obtained showed an increase in half-life and stability [16].

Various PEG sizes were used to determine the effectiveness of the PEGylation method on insulin conformational stability by employing molecular dynamic simulations. The conjugation of PEGs (10-200 ethylene oxide units) to insulin was conducted by an amide bond with the e-amino group of LysB29. The solvent-accessible surface area (SASA) was substantially reduced after conjugation of PEG with insulin, and the PEG-insulin conjugate secondary structure remained unaltered. Furthermore, PEG-protein interactions, such as hydrogen bonds and hydrophobic interaction, beneficially excluded water from the surface of insulin. The PEG length caused all these effects; however, the molecular weight of PEG beyond 4000 Da caused no modifications [5, 17].

Another study previously conducted involved different PEG sizes and demonstrated that the four-unit PEG and PEG with longer chain accelerated folding and reduced unfolding by -0.70+/-0.04 kcalmol^−1^. The PEG with shorter chains imparted less stability towards WW. The WW domain of the human protein Pin 1 referred to as WW protein is mainly used as a model to conduct studies. It was preferably selected due to its two folding energetic states, which have been significantly characterized and allowed amino acid substitutions at many locations. The WW protein has 34 residues that assist their preparation via solid-phase synthesis of peptides, thus simplifying the linkage of shorter PEG oligomer at a single location [14]. The PEG consisting four ethylene oxide units was attached at position 19 of a single Asn side chain of the WW domain of the human protein. The orientation of the side chain at position 19: D-Asn determines the stabilization of PEG-based and well sustained in place of L-Asn at this position. However, PEGylation of the D-residue does not affect WW conformational stability. Such a result (orientation-dependent) may indicate the fundamental PEG-protein interactions attained to PEGylated L-Asn, however contradict PEGylated D-Asn [5, 18]. Several methods have been used to substantially increase conformational stability by PEGylating an Asn residue of WW protein within the reverse turn to accelerate folding and slow unfolding. However, PEGylation protects proteins from proteolysis regardless of the short PEG oligomer. Currently, there is no proper explanation involving direct PEG-OH interactions related to the increase of thermodynamic stability; likely, nearby OH groups the more indirect influence, involving the network of hydrogen-bond solvent molecules surrounding the protein may be exerted. Also, the disorganization of water molecules around nearby residues was increased by PEG. Further, it stabilized the entropic in origin, with advantageous increases in entropy compensating for unfavourable increases in enthalpy. Lawrence et al. [13] further reported that WW conformational and proteolytic stabilities are influenced by both 45- and 4-unit PEG, similarly. Most importantly, the structure-based method can accurately predict the Src SH3 domain located within a beta-sheet protein PEGylation and enhance conformational stability [13].

Additionally, the PEG molecular weight was efficiently proven to reduce autolysis and completely increase the stability of chymotrypsin [19]. Chymotrypsin was conjugated with a different molecular weight of PEG-poly (sulfobetaine methylacrylamide)-block-poly (N-isopropylacrylamide) (pSBAm-block-pNIPAM) (232, 354, and 553 kDa), attempting to elevate pH and thermal stability of chymotrypsin, dramatically. Conjugates and native chymotrypsin were incubated at 37°C for eight hours; conjugates remained stable, while native chymotrypsin lost its 50% initial activity. Similarly, in the incubation of 167 mM HCl for three hours, the native enzyme lost 50% of its activity within 30 minutes followed by destabilization after two hours of all activity, while conjugates showed residual activity of 60% [19]. The positional conjugation of WW at 23 with an azido-functionalized four-unit PEG to a propargyloxyphenylalanine residue normally occupied by Tyr, conformational stability increase of PEGylated protein was seen. Also, it was reported that PEG could subsequently increase the strength of the close by salt-bridge. However, such efficiency is not globally recognized. Its specific structural prerequisites does not involve simple function of secondary structural context, orientation, and distance between PEGylation site and salt bridge, or salt-bridge residue identity [5, 20].

## 3. Site-Specific Protein PEGylation Strategies

Mono-PEGylated and Tri-PEGylated SH3 variant were produced through alkylation of the N-terminal a-amine with the same PEG-aldehyde. Tri-PEGylated SH3 was 0.93 kcal/mol more stable than the non-PEGylated counterpart. PEGylation at the N-terminal did not substantially change the stability of SH3 [21]. Residue specific PEGylation was shown to strengthen the Glu12-Arg14 salt-bridge by shielding it from the interference of water molecules [22]. Recently, Zuma and colleagues (2022) recently demonstrated that site-specific PEGylation enhances the biological activity and stability of recombinant DNA ligase proteins [23]. Cooper and colleagues showed that proteins treated with mono-sulfone-PEG retained higher significantly conjugation [24].

Draper et al. [22] recently reported that specific modification of Asn residue on the side chain amide nitrogen within the WW domain with a 190 kDa monomethoxyPEG greatly enhanced WW conformational and proteolytic stability. In this case, the optimal increase in proteolytic stability was linked with a conformational stability high increase. Furthermore, they found the (identity dependent linker between PEG and protein) alternative PEGylation strategies which effectively alters the WW protein conformational stability [22]. The location, length of PEG, and chemistry used to connect PEG with proteins may influence conformation stability.

## 4. Targeting Cysteine

**Figure 3:**
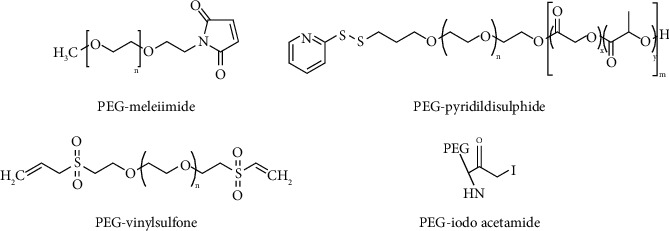
PEGs reactive towards a thiol group [28].

Cysteine residues are mostly covered within the protein structure with low apparition frequency, thus, making them the most interesting targets for residue-specific modification and reduces cysteine from being accessible to chemical reagents. Furthermore, since it is regarded as rare in nature; therefore, they are regularly introduced through genetic engineering [1, 25]. Native chemical ligation (NCL) has been exploited to modify proteins with N-terminal cysteine; this process firstly and reversibly forms a thioester intermediate, followed by a spontaneous shift of S-to-N acyl and end with a production of amide bond [26]. This method was used in PEGylation of HSA molecule-free Cy34 with PEG-maleimide for the protein sulfhydryl (-SH) groups (highly specific) (Figure 3) [16, 27]. Cooper and colleagues showed that proteins treated with mono-sulfone-PEG retained higher significantly conjugation [24]. Dozier and Distefano used this method by PEGylating L-lacate oxidase which retained activity after PEGylation. They mutated serine residue since it was believed to be more susceptible to maleimide PEG, and the results showed a 30% reduction of the activity. Meanwhile, the PEGylated and unmodified mutant presented a decrease of approximately 2.5-folds of resistance to enzymatic activity compared to the wild types [8].

## 5. Targeting Serine, Threonine, and Tryptophan

Targeting the N-terminal position of serines and threonine can generate a glyoxylyl group by utilizing a periodate oxidation reaction which can be used in several linkage formations. This reaction is influenced by the proneness of 1, 2-amino alcohols to periodate oxidation. Previously, Gaertner and Offord (1996) employed a site-specific PEGylation method on N-terminal residue of serine, and sodium perioxidate was used for oxidation and conducted oxime ligation with aminooxy and hydrazide PEG derivative [29]. After PEGylation, the modified proteins (interleukin- (IL-) 8, granulocyte colony-stimulating factor (G-CSF), and IL-1r*α*) retained their biological activity [30]. The direct polymer conjugation of tyrosine residue PEGylation was firstly described by [31]. The three components of tyrosine residue modification such as Mannich-type reaction, coupling with diazonium reagents, and alkylation at the residue were reported to be the most efficient strategies for tyrosine targeting [32]. Recently, Mannich-type reaction modification and reactive coloration in fibrous proteins were done, thus confirming their future applications for the reactive process of silk. The Pictet-Spengler reaction with an aldehyde in glacial acetic acid may be utilized to alter peptides with N-terminal tryptophan residues. This reaction involves N-terminal amino group oxidation to imine, and the cyclic condensation occurs on an aldehyde with the *α*-amine and the indole side chain of a tryptophan residue, leading to the development of a stable C-C bond in a single step [33]. Turecek et al. and Belén et al. employed the Pictet Spengler reaction to label the N-terminal of horse heart myoglobin with an N-terminal glycine by using tryptophan methylester and tryptamine as a linker [4, 16].

## 6. Limitations of PEGylation

Protein PEGylation has been on the market for over 30 years and is the most broadly used post modification technology with structural drawbacks. It has been changing from first-generation to second-generation (which is currently used), and there are new attempts of employing third-generation aiming to increase efficacy. PEG polymers size and position towards conjugates can effectively affect properties. Other drawbacks of PEG include dispersity index, site-specificity of PEG, and PEGylation degree [34]. The polydispersity of PEG may cause challenges, similarly to dispersity towards PEG conjugates. Additionally, the process mainly preventing accessibility of proteolytic enzymes from disrupting PEGylated protein can further prevent accessibility of a substrate from reaching the protein active-site. Therefore, to prevent such complications and eliminate other problems, the active-site protecting agents are used. However, PEGylation around the protected site can still occur. Another method was developed requiring proper pH and ionic fortitude; this process involved the utilization of an inhibitor linked with an insoluble resin (agarose). This was shown to effectively protect the active sites as well as its surroundings. After removing the inhibitor, the biological activity continued to be retained by towards substrates including albumin and blood clots (with urokinase). The PEGylation therapies have caused side effects on patients by entering vasculature and caused hands and foot syndrome (HFS), mucositis, and rash [34]. Other drawbacks have been observed in biotechnology and nanomedicine applications where the receptor binding is decreased due to steric hindrance imposed by the PEG chain's disorder [35]. Enzymes such as cytochrome P450 and alcohol dehydrogenase can gradually reduce the chain length in the *in vivo* experiments. Currently, the known highest PEG molecular weights used for protein conjugation are the 40 kDa branched form [36].

## 7. Alternative Approaches Employed for Recombinant Protein Enhancement

### 7.1. Coexpression Studies

Coexpression is another strategy employed to enhance protein stability, solubility, and bioactivity [3, 4]. The use of chaperone systems such as GroEL-GroES and DnaK-DnaJ-GrpE, or the coexpression of proteins in the presence of trigger factors has been shown to enhance protein solubility [37]. Chaperones specifically favour the solubility of target proteins, thus, coexpression systems tend to favour the solubility of target proteins. Soluble expression (in *E. coli)* of the bacteriophage T4 gene 23 product (major capsid protein) was shown be enhanced by the coexpression of gene product 31 (phage co-chaperonin gp31) [38]. Alternatively, protein production yield, solubility, and folding can be improved through fusion partners or tags (Table 1) [3, 4].

### 7.2. Affinity Tags

Protein fusion has been approved by United States Food and Drug Administration (FDA) for use over 30 years now [39]. Affinity tags are the long-standing tradition for recombinant protein purification, and they have been used to improve protein yield, prevent proteolysis, and increase solubility. Furthermore, fusion partners can translocate passenger protein into different locations with less number of proteases within the cell, thus protecting the produced proteins from degradation. Such fusion partners are maltose-binding protein (MBP) and small ubiquitin-related modifier (SUMO) which could move target protein in the cytosol of *E. coli* to membrane and nucleus, respectively. The MBP and N-utilizing substance A (NusA) are also among the potent solubility-enhancing proteins [40]. Many proteins produce insoluble inclusion bodies during bacterial expression, and only a limited amount (25%) of soluble protein is produced. Therefore, fusion tags are introduced into the recombinant construct when *E. coli* is used thus enhance protein solubility [41]. Alternatively, some tags can be used in the production of toxic proteins; the cellulose-binding modules can be used as a fusion partner in the production of antimicrobial peptides (AMPs) [40].

### 7.3. Use of Nanotechnology to Enhance Proteins

Methods such as encapsulation of proteins within microparticles, chemical modifications with hydrophilic polymers, and recombinant protein engineering have been proven to improve protein therapeutic efficacy. One way to stabilize proteins is to encapsulate the enzymes into nanometer-sized vesicles [42]. This method is employed to protect them from self-denaturation resulting from dilution effects and moreover shield the enzyme from the hostility by external agents like proteases [43]. Outside its stabilizing effect, enzymes encapsulation also adds benefits to biotechnological applications such as manipulation of specificity and molecules delivery for treatment of malignancy.

### 7.4. Liposomes

Liposomes are cost-effective colloidal vesicles ranging from nanometers to a few micrometer thickness, consisting of one or more lipid bilayers surrounding a hydrophilic core. The approval of the use of liposomes as carrier drugs by the US FDA was mainly due to their biocompatibility [57]. Liposomes are excellent vehicles due to their ability to encapsulate hydrophilic substances in the hydrophilic core or hydrophobic substances in space between lipid bilayer. The liposome surfaces possess amenability to be modified with specific moiety for targeted delivery and with biocompatible polymer, such as PEG [58]. The ability of liposomal system to confine enzymes without chemical modifications is beneficial in preserving the inherent enzyme affinity to the cofactor and substrate molecules (Table 1) [58]. To date, liposomes have been employed in development of diagnostic and biosensor materials, functional drugs, and biocompatible catalysts [43].

## 8. Conclusion and Future Recommendations

This review highlighted the importance of protein PEGylation and how the conjugation of protein and PEG increases protein stability. Additionally, the integrated results of enhancing protein stability and other most important factors such as solubility, protein folding, and biological activity and increased the half-life were highlighted in this document. The two known methods for PEGylation have been shown to harbour some advantages and limitations. Nanomaterials and coexpression systems have reviewed a viable alternative to enhance desirable factors in proteins. The application of nanomaterials in therapeutic studies has been widely employed for protein delivery in the treatment and diagnostics. The combined employment of PEGylation, coexpression, and nanosystems for enhancing protein attributes is yet to be explored and may yield desirable protein characteristics such as increased stability and biological activity.

## Figures and Tables

**Table 1:** Summary of protein enhancement systems and their benefits.

Protein enhancement system(s)	Subsystems	Benefits
Protein PEGylation	(i) Covalent (ii) Noncovalent	↑Solubility [44] ↑Aggregation [2] ↓Toxicity [45] ↓Immunogenicity [46] ↑Half-life [47] ↑Thermal stability [23]

Coexpression	(i) Chaperone molecules	↑Folding [37] ↓Aggregation [48] ↑Costability [49] ↑Protein interactions [50] ↑Biological activity [51] ↑Thermal stability [48] ↑Solubility [51]

Protein encapsulation	(i) Liposomal encapsulation	↑Efficacy [52] ↓Cost [53] ↓Immunogenicity [54] ↑Stability [55] ↑Permeability [43] ↑Specificity [56]
